# Factors associated with vertical transmission of HIV in the Western Cape, South Africa: a retrospective cohort analysis

**DOI:** 10.1002/jia2.26235

**Published:** 2024-03-25

**Authors:** Kim Anderson, Emma Kalk, Alexa Heekes, Florence Phelanyane, Nisha Jacob, Andrew Boulle, Ushma Mehta, Reshma Kassanjee, Gayathri Sridhar, Leigh Ragone, Vani Vannappagari, Mary‐Ann Davies

**Affiliations:** ^1^ Centre for Infectious Disease Epidemiology and Research School of Public Health Faculty of Health Sciences University of Cape Town Cape Town South Africa; ^2^ Health Intelligence, Western Cape Department of Health Cape Town South Africa; ^3^ Division of Public Health Medicine School of Public Health Faculty of Health Sciences University of Cape Town Cape Town South Africa; ^4^ ViiV Healthcare Durham North Carolina USA; ^5^ Department of Epidemiology Gilling School of Public Health University of North Carolina Chapel Hill North Carolina USA

**Keywords:** infant, pregnancy, breastfeeding, HIV acquisitions, vertical transmission, antiretroviral therapy

## Abstract

**Introduction:**

Monitoring mother‐infant pairs with HIV exposure is needed to assess the effectiveness of vertical transmission (VT) prevention programmes and progress towards VT elimination.

**Methods:**

We used routinely collected data on infants with HIV exposure, born May 2018–April 2021 in the Western Cape, South Africa, with follow‐up through mid‐2022. We assessed the proportion of infants diagnosed with HIV at birth (≤7 days), 10 weeks (>1 to 14 weeks) and >14 weeks as proxies for intrauterine, intrapartum/early breastfeeding and late breastfeeding transmission, respectively. We used mixed‐effects Poisson regression to assess factors associated with VT in mothers known with HIV by delivery.

**Results:**

We included 50,461 infants born to mothers known with HIV by delivery. HIV was diagnosed in 894 (1.8%) infants. Among mothers, 51% started antiretroviral treatment (ART) before and 27% during pregnancy; 17% restarted during pregnancy after ≥6 months interruption; and 6% had no recorded ART during pregnancy. Most pregnancy ART regimens included non‐nucleoside reverse transcriptase inhibitors (83%). Of mothers with available results (90% with viral load [VL]; 70% with CD4), VL nearest delivery was <100 copies/ml in 78% and CD4 count ≥350 cells/μl in 62%. HIV‐PCR results were available for 86%, 67% and 48% of eligible infants at birth, 10 weeks and >14 weeks. Among these infants, 0.9%, 0.4% and 1.5% were diagnosed positive at birth, 10 weeks and >14 weeks, respectively. Among infants diagnosed with HIV, 43%, 16% and 41% were diagnosed at these respective time periods. Among mothers with VL<100, 100–999, 1000–99,000 and ≥100,000 copies/ml nearest delivery, infant HIV diagnosis incidence was 0.4%, 2.3%, 6.6% and 18.4%, respectively. Increased VT was strongly associated with recent elevated maternal VL with a seven‐fold increased rate with even modestly elevated VL (100–999 vs. <100 copies/ml). VT was also associated with unknown/low maternal CD4, maternal age <20 years, no antenatal ART, later maternal ART start/restart in pregnancy and ART gaps.

**Conclusions:**

Despite high maternal ART coverage and routine postnatal prophylaxis, ongoing VT remains a concern. Timing of infant HIV diagnoses suggests intrapartum and/or breastfeeding transmission in nearly 60%. Interventions to ensure retention on ART and sustained maternal viral suppression are needed to reduce VT.

## 1 INTRODUCTION

Breastfeeding is critical for child health, development and survival, and WHO strongly recommends that in the context of universal maternal antiretroviral treatment (ART), mothers with HIV should breastfeed for ≥12 months [1, 2]. Nevertheless, breastfeeding accounts for >50% of HIV vertical transmission (VT) across 21 priority countries [3]. In a meta‐analysis of postnatal VT after age 4–6 weeks, the pooled estimate of postnatal VT was 1.1% (95% CI 0.3–1.9) by age 6 months and 2.9% (95% CI 0.7–5.2) by 12 months [4]. Although breastfeeding transmission incidence is low, it is not negligible, and in settings with high maternal HIV prevalence, the number of infant acquisitions due to breastfeeding continues to exceed the elimination of VT (eVT) target of <50 new acquisitions/100,000 live births [5]. Rates may be considerably higher in settings with substantial challenges with maternal retention in care, ART adherence and maintenance of viral suppression [6, 7, 8, 9].

There are limited data from routine‐care, resource‐limited settings on risk factors associated with VT [7, 10]. Such data are important to assess programme implementation and determine estimated infant numbers acquiring HIV, national/global estimates of children with HIV and progress towards eVT. As one of 21 countries home to 90% of pregnant women with HIV, South Africa has made great strides in VT prevention [11] and is on the pathway to eVT. South Africa has achieved the WHO target of <5% transmission at breastfeeding cessation but case rates remain 5–10 times higher than the eVT target [5, 12, 13]. Accurate assessment of breastfeeding transmission has been challenging without unique identifiers to allow linking of mother‐infant pairs and evaluation of longitudinal infant testing [14]. Context‐relevant, real‐world evidence of ongoing VT drivers is vital to inform the choice and implementation of targeted interventions to close remaining VT gaps [15, 16]. We used routinely collected data on infants with HIV exposure to assess infant HIV‐testing coverage, evaluate infants diagnosed with HIV at different ages and examine risk factors associated with VT.

## 2 METHODS

Data extraction was facilitated by the Western Cape Provincial Health Data Centre (PHDC), a health information exchange which links individuals via unique identifiers to a range of datasets, including outpatient visits, pharmacy records, laboratory results and hospitalizations [17, 18] Based on these integrated data, health conditions, including HIV, are inferred for individuals. At birth, unique identifiers are issued to infants and linked to maternal identifiers, enabling mother‐infant data linkage. We included infants born 1 May 2018–30 April 2021 in the Western Cape province to mothers with PHDC evidence of HIV prior to/at delivery (Group 1). Separately, we identified additional infants, born during the same 3‐year period, with evidence of HIV acquisition but born to mothers with first HIV evidence after delivery (Group 2) as well as unlinked infants (Group 3; no maternal data). Extraction of a de‐identified, linked dataset was performed on 15 August 2022 (database closure). The study was approved by the Western Cape Department of Health Provincial Health Research Committee and Human Research Ethics Committee at the University of Cape Town (HREC REF 145/2021; waiver of consent included).

### 2.1 Maternal guidelines and definitions

Routine maternal HIV testing is offered at the first antenatal visit. Repeat testing is recommended at ∼20 and 32 weeks gestation, delivery, 6 weeks after delivery and every 3 months while breastfeeding [19, 20, 21]. Newly diagnosed pregnant/breastfeeding women initiate ART on the same day as HIV diagnosis. CD4 cell count is measured at diagnosis and 12 months after ART start; CD4 monitoring thereafter is stopped if CD4≥ 200 cells/μl. At the start of the study period, guidelines recommended measuring viral load (VL) of those on ART at the first antenatal visit. For those starting/restarting ART during pregnancy, VL was measured after 3 months; with closer monitoring of unsuppressed VL in the third trimester. Postpartum VL was measured at 6 months after the previous antenatal VL, and thereafter every 3 months during breastfeeding [20]. In early 2020, guidelines changed to introduce VL testing for all on ART at delivery and 6 months after delivery; repeating VL every 6 months while breastfeeding [21]. We defined viral suppression as VL<100 copies/ml (due to variations in laboratory‐reported detection thresholds, with a range of <20 to <100 copies/ml reported as undetectable). For longitudinal (time‐updated) analyses, “most recent” values were reassigned as “unknown” VL if/while ≥6 months since the previous test and as “unknown” CD4 if/while ≥12 months since the previous test.

During most of the study period, standard first‐line ART for people age ≥15 years (including pregnancy) comprised tenofovir, emtricitabine/lamivudine and efavirenz, as daily fixed‐dose combinations [19]. Standard second‐line ART comprised lopinavir‐ritonavir, lamivudine and zidovudine. Third‐line regimens (determined after genotype resistance testing) may have included darunavir/raltegravir/dolutegravir. In 2020, dolutegravir became the recommended choice for ART‐naïve people initiating ART, ART‐experienced people on first‐line with viral suppression, and second‐line for people failing first‐line non‐nucleoside reverse transcriptase inhibitors regimens [21].

We assessed maternal ART adherence during pregnancy through 12 months postpartum; with ART gaps defined as >2 weeks without drugs per pharmacy dispensing data. We defined mothers as restarting ART if they first started ART before pregnancy but had an interval of ≥6 months without ART and/or an interval of ≥9 months between pharmacy visits (if ART dispensing data were incomplete) before recommencing ART during pregnancy. We regarded mothers as likely having vertically acquired HIV if their first PHDC evidence of HIV occurred at age <12 years.

### 2.2 Infant guidelines and definitions

We calculated the proportion of infants with HIV diagnosis at birth (≤7 days), 10 weeks (8–98 days) and >98 days, as proxies for intrauterine, intrapartum/early breastfeeding and late breastfeeding transmission, respectively. These time periods coincide with routine infant HIV testing. From 2016, infants with HIV exposure received routine HIV‐PCR tests at birth and 10 weeks [19]. At age 9 and 18 months, HIV‐exposed infants received HIV‐Rapid antigen/antibody testing, and testing at 6 weeks after last breastfeed was recommended. Initial positive HIV results (HIV‐PCRs/Rapids) in infants <18 months are confirmed with an HIV‐PCR. At age ≥18 months, positive HIV‐Rapids are confirmed with a second HIV‐Rapid. Testing guidelines changed in early 2020: birth and 10‐week HIV‐PCR testing remained but 9‐month HIV‐Rapids were stopped [21]. Instead, 6‐month HIV‐PCRs were introduced, and all children not known with HIV were to receive universal HIV‐Rapid testing at age 18 months, regardless of exposure status. In our analysis, in the absence of HIV‐PCR data, we inferred HIV acquisition if there was detectable VL at any age, positive HIV‐antigen/antibody/Rapid test at age ≥18 months, age‐appropriate CD4% meeting the criteria for severe immunodeficiency [22] and/or triple ART dispensed repeatedly, without subsequent negative HIV‐PCR/antigen/antibody tests. Because birth HIV‐PCRs are sometimes labelled with mothers’ identifiers (prior to generating infant identifiers), any such negative “maternal” delivery HIV‐PCRs were regarded as of infant origin. Infants with HIV exposure are routinely given postnatal prophylaxis (PNP) as daily nevirapine for 6 weeks (low‐risk cases) or dual nevirapine and zidovudine for ≥6 weeks (high‐risk cases). Infants with an increased risk of HIV transmission during breastfeeding remain on nevirapine until maternal VL <1000 copies/ml [19, 20, 21].

### 2.3 Data analysis

We described characteristics of mother‐infant pairs using counts and proportions or medians with interquartile ranges (IQRs), as appropriate (all groups). Among infants whose mothers were known with HIV by delivery (Group 1), we calculated the proportion of infants diagnosed with HIV at different time intervals, with the denominator being those not previously testing positive and with an available test at each interval. We calculated unadjusted and adjusted incidence rate ratios (aIRR) for VT using Poisson and mixed‐effects Poisson regression models, respectively (Group 1). We excluded twins/triplets from regression models. Because some mothers had more than one child in the study period (i.e. non‐twin siblings), we incorporated random effects to account for the clustering of infants by mother. We considered infant sex, birthweight, infant PNP, year of birth, maternal age, whether the mother had vertically acquired HIV herself, parity, timing of maternal ART start, ART gaps during pregnancy, and most recent maternal CD4 and VL as factors potentially associated with VT *a priori*. We assessed associations with infant HIV diagnosis at different ages in separate models, as they represent potentially different routes of transmission. Infants were censored at death or database closure. We assumed that untested infants were uninfected. In case this assumption resulted in misclassification of infant HIV status or timing of acquisition, we performed sensitivity analyses including: (a) only infants who had a negative test in the previous period and were tested in the current period of analysis; and (b) an alternative composite outcome of death/HIV diagnosis. We expected breastfeeding rates and VT rates/diagnosis to decrease as infants aged, therefore, we included an age category covariate in models assessing late breastfeeding transmission.

## 3 RESULTS

We included 50,461 HIV‐exposed infants born to 48,166 mothers known with HIV by delivery (Group 1). Of these infants, 894 (1.8%) overall were diagnosed with HIV. We identified an additional 702 infants with HIV, born during the same period: 303 to mothers whose first HIV evidence was after delivery (Group 2) and 399 for whom maternal data were unavailable (Group 3). The proportion of Group 1 infants with HIV diagnosis at different time intervals, as a proxy for different routes of transmission, was 0.9%, 0.4% and 1.5% at age ≤7, 8–98 and >98 days, respectively (including only those not previously testing positive and with an available test/diagnosis at each interval in the denominator).

Tables 1 and S1 show mother‐infant characteristics stratified by group.

**Table 1:** Infant and maternal characteristics, stratified by group

		Total	Group 1	1A	1B	Group 2	Group 3
			Child HIV exposed, maternal HIV evidence ≤delivery date	Child HIV negative, exposed to HIV	Child with HIV	Child with HIV, maternal HIV evidence >delivery date	Child with HIV, no maternal linkage
Total number of infants		51,163	50,461	49,567	894	303	399
Number of mother‐infant pairs with linked data (*n* = 51,163)		50,764 (99.2%)	50,461	49,567	894	303	0
Number of infants with evidence^a^ of HIV (*n* = 51,163 [50,461; 303; 399])		1596 (3.1%)	894 (1.8%)	0	894	303 (100%)	399 (100%)
Male sex (infant) (*n* = 51,120 [50,418; 303; 399])		25,743 (50.4%)	25,393 (50.4%)	24,948 (50.4%)	445 (49.8%)	141 (46.5%)	209 (52.4%)
Year of infant birth (*n* = 51,163 [50,461; 303; 399])	Year 1 (01/05/2018–30/04/2019)	16,242 (31.7%)	15,957 (31.6%)	15,624 (31.6%)	333 (34.7%)	115 (38.0%)	170 (42.6%)
	Year 2 (01/05/2019–30/04/2020)	17,123 (33.5%)	16,878 (33.4%)	16,570 (33.5%)	308 (32.0%)	101 (33.4%)	144 (36.1%)
	Year 3 (01/05/2020–30/04/2021)	17,798 (34.8%)	17,626 (34.9%)	17,306 (35.0%)	320 (33.3%)	87 (28.7%)	85 (21.2%)
Number of infant deaths (*n* = 51,163 [50,461; 303; 399])		763 (1.5%)	737 (1.5%)	693 (1.4%)	44 (4.9%)	12 (4.0%)	14 (3.5%)
Number of maternal deaths, per infant (*n* = 50,764 [50,461; 303])		483 (1.0%)	482 (1.0%)	440 (0.9%)	42 (4.7%)	1 (0.3%)	NR
Number of live‐birth infants from multiple pregnancies (twins/triplets) (*n* = 50,764 [50,461; 303])		1672 (3.3%)	1668 (3.3%)	1644 (3.3%)	24 (2.7%)	4 (1.3%)	NR
Maternal age at delivery (years), per infant (median; IQR) (*n* = 50,764 [50,461; 303])		30.4 (26.1–34.6)	30.4 (26.1–34.7)	30.5 (26.2–34.7)	28.8 (24.9–32.8)	26.3 (22.4–29.8)	NR
Birthweight in grams (median; IQR) (*n* = 50,538 [50,241; 297])		3060 (2680–3400)	3060 (2680–3400)	3060 (2690–3400)	2835 (2385–3190)	2960 (2630–3335)	NR
Low birthweight (<2500 g) (*n* = 50,538 [50,241; 297])		8629 (17.1%)	8569 (17.1%)	8299 (16.8%)	270 (30.6%)	60 (20.2%)	NR
Very low birthweight (<1500 g) (*n* = 50,538 [50,241; 297])		1453 (2.9%)	1447 (2.9%)	1409 (2.9%)	38 (4.3%)	6 (2.0%)	NR
Infant born before arrival at a health facility (vs. born in a health facility) (*n* = 50,750 [50,450; 300])		975 (1.9%)	963 (1.9%)	923 (1.9%)	40 (4.2%)	12 (4.0%)	NR
Infant prophylaxis dispensed^b^ in week 1 of life (*n* = 51,163 [50,461; 303; 399])		23,356 (45.7%)	23,335 (46.2%)	22,983 (46.4%)	352 (39.4%)	9 (3.0%)	12 (3.0%)
Maternal parity^c^ (*n* = 50,745 [50,448; 297])	0 (primiparous)	23,766 (46.8%)	23,594 (46.8%)	23,183 (46.8%	411 (46.6%)	172 (57.9%)	NR
	1	16,193 (31.9%)	16,118 (32.0%)	15,863 (32.0%)	255 (28.9%)	75 (25.3%)	NR
	≥2	10,786 (21.3%)	10,736 (21.3%)	10,520 (21.2%)	216 (24.5%)	50 (16.8%)	NR
Maternal HIV diagnosed before age 12 years (proxy for vertically acquired HIV), per infant (*n* = 50,461)		292 (0.6%)	292 (0.6%)	282 (0.6%)	10 (1.1%)	N/A	NR
Timing of maternal ART initiation, per infant (*n* = 50,764 [50,461; 303])	Before pregnancy	25,492 (50.2%)	25,492 (50.5%)	25,227 (50.9%)	265 (29.6%)	0	NR
	During pregnancy	13,573 (26.7%)	13,573 (26.9%)	13,290 (26.8%)	283 (31.7%)	0	NR
	Restarted^d^ during pregnancy	8587 (16.9%)	8587 (17.0%)	8312 (16.8%)	275 (30.8%)	0	NR
	After pregnancy (>delivery date)	852 (1.7%)	651 (1.3%)	612 (1.2%)	39 (4.4%)	201 (66.3%)	NR
	No ART recorded	2260 (4.5%)	2158 (4.3%)	2126 (4.3%)	32 (3.6%)	102 (33.7%)	NR
Maternal regimen^e^ during pregnancy (<infant DOB), per infant (*n* = 45,035)	NNRTI‐based	37,508 (83.3%)	37,508 (83.3%)	36,953 (83.3%)	555 (80.4%)	0	NR
	PI‐based	2153 (4.8%)	2153 (4.8%)	2093 (4.7%)	60 (8.7%)	0	NR
	INSTI‐based	5031 (11.2%)	5031 (11.2%)	4961 (11.2%)	70 (10.1%)	0	NR
	Unknown	343 (0.8%)	343 (0.8%)	338 (0.8%)	5 (0.7%)	0	NR
Maternal regimen^f^ during first 12 months after delivery, per infant (*n* = 43,427 [43,302; 125])	NNRTI‐based	27,859 (64.2%)	27,785 (64.2%)	27,301 (64.1%)	484 (65.4%)	74 (59.2%)	NR
	PI‐based	2215 (5.1%)	2215 (5.1%)	2135 (5.0%)	80 (10.8%)	0	NR
	INSTI‐based	13,129 (30.2%)	13,083 (30.2%)	12,912 (30.3%)	171 (23.1%)	46 (36.8%)	NR
	Unknown	224 (0.5%)	219 (0.5%)	214 (0.5%)	5 (0.7%)	5 (4.0%)	NR

*Note*: Denominators are indicated as “*n* = total (x; y; z)” where appropriate, with x, y and z representing denominators for groups 1, 2 and 3, respectively (e.g. “*n* = 51,163 (50,461; 303; 399)” indicates that all infants are included, none have missing data).

Abbreviations: ART, antiretroviral therapy; DOB, date of birth; INSTI, integrase strand transfer inhibitor; N/A, not applicable; NNRTI, non‐nucleoside reverse transcriptase inhibitor; NR, not recorded; PI, protease inhibitor; TB, tuberculosis.

^a^ HIV evidence: 1241 infants (78%) with positive HIV‐PCRs; 355 (22%) with either positive serology tests at age ≥18 months, detectable VL at any age, severe immunodeficiency (as per CD4%) and/or ART dispensed repeatedly, without subsequent negative HIV‐PCR/antigen/antibody tests.

^b^ Missing data likely as infant postnatal prophylaxis is not reliably captured electronically by facilities.

^c^ Recorded during the pregnancy and is based on prior digital evidence of pregnancy in the province.

^d^ Based on ART gap > 183 days and/or pharm visit gap >274 days, among infants whose mothers were known to have started ART before pregnancy.

^e^ Prioritized any dispensing of INSTIs over PIs over NNRTIs (when regimens changed), therefore, regimen listed does not necessarily apply for the entire duration.

^f^ Started ever before pregnancy (not necessarily ongoing during pregnancy).

**Figure 1:**
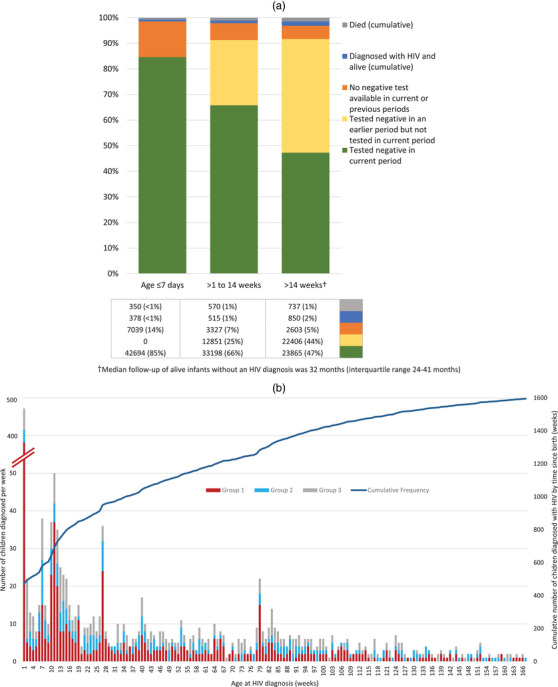
(a) Cumulative vital status and HIV status at different time intervals for infants with HIV exposure (Group 1 infants, mothers known with HIV at delivery; *n* = 50,461) and (b) age in weeks at first evidence of HIV in infants, by group, shown up to age 3 years (Group 1 are infants whose mothers were known with HIV before/at delivery; Group 2 are infants whose mothers’ first HIV evidence was after delivery; and Group 3 are infants with no maternal linkage, i.e. timing of maternal HIV unknown). †Median follow‐up of alive infants without an HIV diagnosis was 32 months (IQR 24–41 months).

Figure 1a shows vital status and HIV status at different time intervals (Group 1). During the day 8–98 interval, excluding deceased infants and those known with HIV, a third of infants assumed alive and uninfected had unverifiable HIV status (either tested negative at birth but not tested in the current interval [26%] or no test at birth or current interval [7%]), whereas after age 98 days, half (51%) had unverifiable HIV status, although HIV‐Rapid testing data were likely incomplete as HIV‐Rapid results are not routinely captured electronically by all facilities. Figure 1b shows age at diagnosis of HIV in all infants and Figure S1 shows the number of HIV tests performed in Group 1 infants by age. Observed peaks in testing and HIV diagnoses align with programmatic HIV testing at birth, 10 weeks and 6 months, especially at birth.

Table 2 shows HIV‐related mother‐infant laboratory results by group. Among Group 1 mothers with available results, 62% had CD4≥350 cells/μl and 78% had VL<100 copies/ml nearest delivery. Among mothers with VL<100, 100–999, 1000–99,000 and ≥100,000 copies/ml nearest delivery, the incidence of infant HIV diagnosis was 0.4%, 2.3%, 6.6% and 18.4%, respectively. HIV‐PCR testing coverage was 86%, 67% and 48% among Group 1 infants at age ≤7, 8–98 and >98 days, respectively. Of infants diagnosed with HIV (all groups), 30%, 19% and 51% were diagnosed at these respective time periods.

**Table 2:** A) Maternal and (B) infant HIV‐related laboratory results, stratified by group

		TOTAL	Group 1	1A	1B	Group 2	Group 3
(A) Maternal results, per linked infant			Child HIV exposed, maternal HIV evidence ≤delivery date	Child HIV‐negative, exposed to HIV	Child with HIV	Child with HIV, maternal HIV evidence >delivery date	Child with HIV, no maternal linkage
Number of mother‐infant pairs with linked data		50,764 (100%)	50,461 (99.4%)	49,567 (97.6%)	894 (1.8%)	303 (0.6%)	0
Maternal immune deficiency category nearest to delivery^a^, per infant (CD4 count; cells/μl) (*n* = 35,310 [35,289; 21])	No deficiency (≥500)	13,425 (38.0%)	13,421 (38.0%)	13,317 (38.5%)	104 (14.3%)	4 (19.0%)	
	Mild (350–499)	8604 (24.4%)	8596 (24.4%)	8454 (24.5%)	142 (19.6%)	8 (38.1%)	
	Advanced (200–349)	8326 (23.6%)	8323 (23.6%)	8119 (23.5%)	204 (28.1%)	3 (14.3%)	
	Severe (<200)	4955 (14.0%)	4949 (14.0%)	4673 (13.5%)	276 (38.0%)	6 (28.6%)	
Number of linked infants with no maternal CD4 close to delivery (*n* = 50,764)		15,454 (30.4%)					
Maternal immune deficiency category postnatally (nearest 12 months)^b^, per infant (CD4 count; cells/μl) (*n* = 15,847 [15,666; 181])	No deficiency (≥500)	8113 (51.2%)	8042 (51.3%)	7931 (52.4%)	111 (20.4%)	71 (39.2%)	
	Mild (350–499)	3136 (19.8%)	3086 (19.7%)	3002 (19.9%)	84 (15.5%)	50 (27.6%)	
	Advanced (200–349)	2687 (17.0%)	2651 (16.9%)	2504 (16.6%)	147 (27.1%)	36 (19.9%)	
	Severe (<200)	1911 (12.1%)	1887 (12.0%)	1686 (11.1%)	201 (37.0%)	24 (13.3%)	
Number of linked infants with no postnatal maternal CD4 (*n* = 50,764)		34,917 (68.8%)					
Maternal VL category during pregnancy^c^, nearest to delivery, per infant (copies/ml) (*n* = 45,180 [45,169; 11])	<100		35,382 (78.3%)	35,381 (78.3%)	35,214 (79.3%)	167 (22.6%)	1 (9.1%)
	100–999	4111 (9.1%)	4111 (9.1%)	4008 (9.0%)	103 (13.9%)	0	
	1000–99,999	4960 (11.0%)	4953 (11.0%)	4618 (10.4%)	335 (45.3%)	7 (63.6%)	
	≥100,000	727 (1.6%)	724 (1.6%)	590 (1.3%)	134 (18.1%)	3 (27.3%)	
Number of linked infants with no maternal pregnancy VL (*n* = 50,764)		5584 (11.0%)	5292 (10.5%)	5137 (10.4%)	155 (17.3%)	292 (96.4%)	
VL at delivery^d^ category (copies/ml) (*n* = 26,369 [26,358; 11])	<100	19,665 (74.6%)	19,664 (74.6%)	19,577 (75.8%)	87 (16.9%)	1 (9.1%)	
	100–999	2440 (9.3%)	2440 (9.3%)	2373 (9.2%)	67 (13.0%)	0	
	1000–99,999	3682 (14.0%)	3675 (13.9%)	3425 (13.3%)	250 (48.5%)	7 (63.6%)	
	≥100,000	582 (2.2%)	579 (2.2%)	468 (1.8%)	111 (21.6%)	3 (27.3%)	
Number of linked infants with no maternal delivery VL (*n* = 50,764)		24,395 (48.1%)	24,103 (47.8%)	23,724 (47.9%)	379 (42.4%)	292 (96.4%)	
Postpartum (nearest 6 months)^e^ VL category (copies/ml) (*n* = 29,777 [29,696; 81])	<100	25,346 (85.1%)	25,294 (85.2%)	25,119 (85.9%)	175 (37.6%)	52 (64.2%)	
	100–999	1715 (5.8%)	1710 (5.8%)	1642 (5.6%)	68 (14.6%)	5 (6.2%)	
	1000–99,999	2309 (7.8%)	2287 (7.7%)	2141 (7.3%)	146 (31.4%)	22 (27.2%)	
	≥100,000	407 (1.4%)	405 (1.4%)	329 (1.1%)	76 (16.3%)	2 (2.5%)	
Number of linked infants with no postpartum maternal VL (*n* = 50,766)		20,987 (41.3%)	20,765 (41.2%)	20,336 (41.0%)	429 (48.0%)	222 (73.3%)	

(B) Infant results		TOTAL	Group 1	1A	1B	Group 2	Group 3
Number of exposed infants		51,163	50,461	49,567	894	303	399
Number of infants with any HIV‐PCR test (*n* = 51,163)		47,750 (93.3%)	47,262 (93.7%)	46,382 (93.6%)	880 (98.4%)	235 (77.6%)	253 (63.4%)
Number of infants with a positive PCR, among those tested (*n* = 47,750 [47,262; 235; 253])		1241 (2.6%)	776 (1.6%)	0	776 (88.2%)	229 (97.4%)	236 (93.3%)
Median age at first positive PCR (days) (IQR) (*n* = 1241 [776; 229; 236])		75 (1–276)	42 (0–186)	N/A	42 (0–186)	163 (60–388)	105 (10–337)
Median age at first evidence of HIV (days) (IQR) (*n* = 1596)		106 (1–450)	70 (0–307)	N/A	70 (0–307)	254 (74–602)	193 (50–560)
Number of infants without a PCR, among those who died age ≤7 days (*n* = 350)		184 (52.6%)	184 (52.6%)			N/A	N/A
Number of infants with combined evidence^f^ of a birth PCR (at ≤7 days old) (*n* = 51,167 [50,461; 303; 399])		43,417 (84.9%)	43,293 (85.8%)			48 (15.8%)	76 (19.0%)
Number of infants^g^ with a PCR at week 1–14 (day 8–98) (*n* = 50,314 [49,702; 270; 342])		33,518 (66.6%)	33,363 (67.1%)			67 (24.8%)	88 (25.7%)
Number of infants^g^ with both a birth PCR and week 1–14 PCR (*n* = 50,314 [49,700; 270; 342])		29,746 (59.1%)	29,723 (59.8%)			10 (3.7%)	13 (3.8%)
Number of infants^h^ with a PCR at >98 days (*n* = 49,782 [49,327; 205; 250])		23,909 (48.0%)	23,640 (47.9%)			139 (67.8%)	130 (52.0%)
Age at infant diagnosis of HIV (*n* = 1596 [894; 303; 399])	≤7 days (“birth”)	472 (30.3%)	382 (42.7%)			33 (10.9%)	57 (14.3%)
	day 8–98 (“10 weeks”)	303 (19.0%)	146 (16.3%)			65 (21.5%)	92 (23.1%)
	99–365 days	351 (22.0%)	168 (18.8%)			83 (27.4%)	100 (25.1%)
	>365 days	470 (29.4%)	198 (22.1%)			122 (40.3%)	150 (37.6%)
Number of infants with first HIV evidence at week 1–14 who had a previous negative birth PCR (*n* = 303 [146; 65; 92])		111 (36.6%)	102 (69.9%)		102 (69.9%)	4 (6.2%)	5 (5.4%)
Number of infants with first evidence of HIV age >98 days who had a previous negative PCR at 1–14 weeks (*n* = 821 [366; 205; 250])		244 (29.7%)	208 (56.8%)		208 (56.8%)	7 (3.4%)	29 (11.6%)
Number of infants with first evidence of HIV age >98 days who had a previous negative birth PCR (*n* = 821 [366; 205; 250])		334 (40.7%)	308 (84.2%)		308 (84.2%)	12 (5.9%)	14 (5.6%)
Number of infants with first evidence of HIV age >98 days who had a previous negative birth PCR and negative PCR at 1–14 weeks (*n* = 821 [366; 205; 250])		188 (22.9%)	177 (48.4%)		177 (48.4%)	5 (2.4%)	6 (2.4%)

*Note*: Denominators are indicated as “*n* = total (x; y; z)” where appropriate, with x, y and z representing denominators for groups 1, 2 and 3, respectively.

Abbreviations: IQR, interquartile range; VL, viral load.

^a^ Used an interval of 15 months before delivery to 1 week after delivery; and used the CD4 closest to delivery date; median time from delivery (of CD4 closest to delivery) was 133 days (IQR 70–192).

^b^ Used an interval of from >1 week after delivery to 24 months post‐delivery; and used the CD4 closest to 12 months post‐delivery; median time from delivery (of CD4 postnatal measurements) was 370 days (IQR 233–520).

^c^ Used an interval of from 1 month before pregnancy start to 1 week after delivery; and used the VL closest to delivery date; median time from delivery (of pregnancy VL measurement) was 19 days (IQR 1–55).

^d^ Used an interval of from 1 month before to 1 week after delivery; and used the VL closest to delivery date; median time from delivery (of VL at delivery measurement) was 1 day (IQR 0–11).

^e^ Used an interval of from > 1 week to 12 months after delivery; and used the VL closest to 6 months after delivery date; median time after delivery (of postpartum VL measurements) was 184 days (IQR 112–251).

^f^ Includes 1326 infants who did not have a birth PCR on record but there was a negative “maternal” birth PCR (at ≤7 days), which was presumably of infant origin.

^g^ Excluding those with HIV diagnosis at age ≤7 days and those who died at age ≤7 days.

^h^ Excluding those with HIV diagnosis at age ≤98 days and who died at age ≤98 days.

Table S2 shows differences in HIV‐PCR testing coverage by year of infant birth. Birth and 10‐week testing declined (91% vs. 80% and 72% vs. 66%, for infants born in Year 1 vs. 3, respectively). HIV‐PCR testing at age >98 days increased from 28% to 62% (Year 1 vs. 3), attributable to the introduction of programmatic HIV‐PCR testing at 6 months in 2020.

### 3.1 Factors associated with VT

Tables S3 and 3 show findings from univariable and multivariable Poisson analyses (Group 1). Sensitivity analyses are shown in Tables S4 and S5.

**Table 3:** Mixed‐effects Poisson regression models assessing associations with vertical transmission in infants whose mothers were known with HIV by delivery (Group 1 infants)

		Model A (*N* = 47,107 groups)			Model B (*N* = 46,531 groups)			Model C (*N* = 46,269 groups)		
		aIRR	95% CI	*p*	aIRR	95% CI	*p*	aIRR	95% CI	*p*
Infant sex male (vs. female)		0.75	(0.56–1.01)	0.06	0.86	(0.62–1.19)	0.37	1.21	(0.98–1.50)	0.07
Low birthweight (<2500 g) (vs. ≥2500 g)		2.84	(2.03–3.97)	<0.001	1.10	(0.74–1.63)	0.65	1.59	(1.25–2.03)	<0.001
Infant prophylaxis dispensed within week 1 of life (vs. none recorded)		N/A			0.74	(0.52–1.04)	0.08	1.14	(0.93–1.41)	0.21
Maternal age category at delivery (years)	≥ 30 years	1			1			1		
	≥ 20 years and <30 years	1.15	(0.83–1.57)	0.40	1.15	(0.81–1.64)	0.44	1.24	(0.99–1.55)	0.06
	< 20 years	0.97	(0.45–2.08)	0.93	1.13	(0.49–2.63)	0.77	2.11	(1.28–3.51)	0.004
Mother likely acquired HIV vertically (HIV evidence <age 12 years vs. later)		0.15	(0.01–1.96)	0.15	2.27	(0.68–7.66)	0.19	1.67	(0.71–3.93)	0.24
Parity	0 (primiparous)	1			1			1		
	1	0.94	(0.66–1.34)	0.74	0.95	(0.63–1.43)	0.80	1.00	(0.77–1.30)	0.98
	≥2	1.08	(0.71–1.63)	0.72	1.01	(0.64–1.60)	0.96	1.30	(0.98–1.72)	0.07
Detailed categories of antenatal maternal ART (combining ART start timing and ART dispensing gaps of >2 weeks in pregnancy)	Started before pregnancy and no ART gaps during pregnancy	1			1			1		
	Started before pregnancy and had gap/s during pregnancy	3.57	(1.37–9.30)	0.01	2.64	(0.89–7.82)	0.08	2.32	(1.37–3.94)	0.002
	Started before pregnancy but no ART during pregnancy	9.36	(3.52–24.91)	<0.001	3.89	(1.28–11.81)	0.02	9.75	(5.61–16.96)	<0.001
	Started during pregnancy, more than 8 weeks before delivery and no gap/s thereafter during pregnancy	4.35	(1.59–11.91)	0.004	2.70	(0.85–8.59)	0.09	1.73	(0.94–3.20)	0.08
	Started during pregnancy, more than 8 weeks before delivery and had gaps thereafter during pregnancy	6.82	(2.58–18.00)	<0.001	3.99	(1.34–11.93)	0.01	4.38	(2.54–7.56)	<0.001
	Started during pregnancy, but within 8 weeks of delivery	22.62	(8.41–60.87)	<0.001	3.15	(0.94–10.56)	0.06	5.73	(3.06–10.70)	<0.001
	Restarted during pregnancy, more than 8 weeks before delivery and no gap/s thereafter during pregnancy	9.12	(2.57–32.40)	0.001	2.19	(0.40–12.09)	0.37	4.46	(2.15–9.25)	<0.001
	Restarted during pregnancy, more than 8 weeks before delivery and had gaps thereafter during pregnancy	8.41	(3.27–21.64)	<0.001	4.22	(1.46–12.21)	0.01	6.10	(3.67–10.12)	<0.001
	Restarted during pregnancy, but within 8 weeks of delivery	7.07	(2.44–20.42)	<0.001	4.99	(1.56–15.86)	0.01	4.89	(2.50–9.55)	<0.001
	No ART prior to or during pregnancy	9.17	(3.40–24.70)	<0.001	2.90	(0.91–9.27)	0.07	4.44	(2.33–8.46)	<0.001
Time‐updated CD4 immune deficiency category (cells/μl)	No deficiency (≥500)	1			1			1		
	Mild (350–499)	1.65	(1.00–2.74)	0.05	1.22	(0.61–2.42)	0.58	2.48	(1.33–4.63)	0.004
	Advanced (200–349)	1.25	(0.76–2.04)	0.38	1.98	(1.08–3.62)	0.03	4.97	(2.85–8.67)	<0.001
	Severe (<200)	2.61	(1.58–4.32)	<0.001	2.32	(1.26–4.30)	0.01	7.00	(4.02–12.17)	<0.001
	Unknown	0.72	(0.44–1.20)	0.21	1.13	(0.59–2.14)	0.72	3.35	(1.99–5.66)	<0.001
Time‐updated VL (copies/ml)	<100	1			1			1		
	100–999	7.35	(3.98–13.58)	<0.001	7.21	(3.40–15.29)	<0.001	2.46	(1.19–5.07)	0.02
	1000–99,999	38.15	(22.94–63.45)	<0.001	20.20	(10.79–37.82)	<0.001	5.68	(3.46–9.30)	<0.001
	≥100,000	244.75	(121.17–494.36)	<0.001	56.67	(27.89–115.16)	<0.001	25.89	(15.57–43.06)	<0.001
	Unknown	25.94	(15.36–43.83)	<0.001	11.65	(5.95–22.80)	<0.001	6.90	(4.62–10.32)	<0.001
Year of infant birth	Year 1 (01/05/2018–0/04/2019)	1			1			1		
	Year 2 (01/05/2019–30/04/2020)	0.97	(0.68–1.39)	0.86	0.99	(0.66–1.49)	0.96	0.93	(0.72–1.20)	0.57
	Year 3 (01/05/2020–30/04/2021)	1.39	(0.97–2.01)	0.08	1.11	(0.74–1.68)	0.61	0.78	(0.58–1.03)	0.08

*Note*: Adjusted IRRs (and 95% confidence intervals) were obtained from mixed‐effects Poisson regression models (log link function; normally distributed random effect by mother; observation time as an offset). Estimated variance of random effects at maternal level were all < 0.01, suggesting no remaining unexplained inter‐mother variability. Twins and triplets were excluded from analyses. In Model C, an age category covariate was included (age 3–5, 5–8, 8–11, 11–14, 14–17, 17–24 and ≥24 months). Model A assesses associations with HIV diagnosis in infants at age ≤7 days, Model B assesses diagnosis at 8–98 days and Model C assesses diagnosis at age >98 days.

Abbreviations: aIRR, adjusted incidence rate ratio; ART, antiretroviral therapy; CI, confidence interval; VL, viral load.

### 3.2 Intrauterine transmission

In multivariable analysis (Model A, Table 3), low birthweight and low maternal CD4 (CD4<200 vs. CD4≥500 cells/μl) were associated with three times higher intrauterine transmission rates. Timing of ART start and gaps in pregnancy ART were associated with intrauterine transmission. The largest effect was seen for mothers who started ART ≤8 weeks before delivery (23 times higher rates), compared to starting ART before pregnancy without gaps. Restarting ART during pregnancy, starting ART during pregnancy but with gaps thereafter or receiving no ART during pregnancy were associated with 7–9 times higher rates. Unknown and incrementally higher maternal VL were strongly associated with correspondingly higher transmission rates, with VL 100–999, 1000–99,999 and >100,000 copies/ml associated with seven, 38 and 245 times higher rates, respectively, versus VL<100 copies/ml. In sensitivity analyses, excluding infants without birth testing, overall findings were similar (Model D, Table S4).

### 3.3 Early postnatal transmission

In multivariable analysis (Model B, Table 3), lower maternal CD4 was associated with early transmission (two times higher rates if CD4<200 or 200–349, vs. CD4≥500 cells/ μl). Timing of ART start and gaps in antenatal ART were associated with early transmission. Starting ART before pregnancy but with no ART during pregnancy, starting/restarting ART ≤8 weeks before delivery or starting/restarting >8 weeks before delivery but with antenatal gaps thereafter had rates 3–5 times higher than starting ART before pregnancy without gaps. Unknown and incrementally higher maternal VL were strongly associated with correspondingly higher transmission rates, with VL 100–999, 1000–99,999 and >100,000 copies/ml associated with seven, 20 and 57 times higher rates, respectively, versus VL<100 copies/ml. In sensitivity analyses, including only infants with previous negative birth tests who tested in the day 8–98 interval, overall findings were similar (Model E, Table S4).

### 3.4 Late postnatal transmission

In multivariable analysis (Model C, Table 3), low birthweight, younger maternal age at delivery (<20 vs. ≥30 years) and unknown/low maternal CD4 were associated with late (breastfeeding) transmission. CD4 350–499, 200–349 and <200 were associated with transmission rates two, five and seven times higher, respectively, compared to CD4≥500 cells/μl. Timing of ART start and gaps in antenatal ART were associated, with the largest effect seen for mothers who started ART before pregnancy but received no ART during pregnancy (10 times higher rates). Starting/restarting ART during pregnancy ≤8 weeks before delivery, starting/restarting >8 weeks before delivery but with antenatal ART gaps thereafter, or restarting >8 weeks before delivery without ART gaps thereafter had rates 4–6 times higher than starting ART before pregnancy without gaps. Unknown and incrementally higher maternal VL were strongly associated with correspondingly higher transmission rates, with VL 100–999, 1000–99,999 and >100,000 copies/ml associated with two, six and 26 times higher rates, respectively, versus VL<100 copies/ml. In sensitivity analyses, including only infants with negative day 8–98 tests who tested at >98 days, birthweight was not significantly associated, whereas higher parity was; other findings were similar (Model F, Table S4).

## 4 DISCUSSION

Of all infants born within a 3‐year period who were diagnosed with HIV in the province, 30% were diagnosed at birth, while the majority were diagnosed later in life, including 19% at age 10 weeks, 22% between 3 months and 1 year and 29% at age ≥1 year. Of these infants with HIV, 56% were born to mothers known with HIV by delivery (38% before pregnancy), 19% to mothers diagnosed with HIV after delivery and 25% had missing maternal data. Among infants whose mothers were known with HIV by delivery, we found that increased VT was associated with inconsistent maternal ART usage during pregnancy, low maternal CD4 and elevated maternal VL. Concerningly, we found that even a modestly elevated VL (100–999 copies/ml) which would not meet the definition of viral failure [20] was associated with VT rates up to seven times higher than VL<100 copies/ml. This emphasizes the importance of achieving and maintaining undetectable VL during pregnancy and breastfeeding. In all cases of HIV diagnosis at age ≥8 days after a negative birth PCR, where the mother's most recent VL nearest to infant diagnosis was undetectable (within an interval of 3 months before to 2 weeks after diagnosis; *n* = 11), there was evidence of either earlier detectable VL postpartum or ART gaps of >2 weeks in the postpartum period. Increased risk of VT has been documented with low‐level viraemia during pregnancy/postpartum in other studies, although differing VL thresholds were considered as low‐level, including 40–1000 and 50–400 copies/ml [23, 24].

Several studies have shown zero sexual transmission of HIV with VL<200 copies/ml [25] but for the prevention of VT, achieving undetectable VL is essential.

We found that starting ART before pregnancy but not receiving ART during pregnancy or restarting during pregnancy had particularly elevated VT risk, as well as starting ART <2 months before delivery or having ART gaps of >2 weeks during pregnancy. While elevated maternal VL and lower CD4 have previously been shown to be strong predictors of VT, [26, 27] we additionally found that unknown recent VL was associated with higher VT rates, likely reflecting maternal disengagement from care. Younger maternal age was associated with increased late transmission; other studies too have reported poorer viral suppression and adherence [28, 29, 30, 31] with higher VT risk [32] in younger pregnant/postpartum adolescents/women.

Overall, 78% of pregnant women who were tested achieved viral suppression before delivery, but the >20% who did not are a high‐risk group needing intensive support and vigilant mother‐infant monitoring. Mothers who transmit HIV to their infants in the era of universal maternal ART represent a vulnerable population who require improved access to care and enhanced social and adherence support. To close the gaps to achieve eVT in our high HIV prevalence setting, we need to identify and support mothers‐at‐risk and help pregnant and postpartum women achieve and maintain viral suppression as a matter of urgency. It is well‐known, however, that keeping mothers engaged in care and optimally adherent to ART postpartum is challenging [30, 33, 34, 36].

To reduce VT in women who are not yet known with HIV by delivery, regular HIV testing during pregnancy, at delivery and postpartum is required, with provision of pre‐exposure prophylaxis to those who test negative. It has been estimated that failure to detect maternal HIV during pregnancy is responsible for up to 54% of VT in resource‐limited settings [36]. In 2014–2016, delivery testing coverage among women without an HIV diagnosis in Cape Town was only 23%. Although first antenatal visit testing is well implemented (98%), major gaps exist in implementing HIV testing thereafter [36], despite the risk of HIV seroconversion during pregnancy and postpartum being high in our setting and contributing significantly to VT [37, 38, 39].

In the later part of the study period, dolutegravir replaced efavirenz as the preferred third drug for people starting ART. However, the rollout of dolutegravir in South Africa occurred slowly, and women were initially less likely to receive dolutegravir than men [40]. As more women initiated/switched to dolutegravir recently, it is hoped that subsequent VT outcomes may improve. Compared to efavirenz, viral suppression with dolutegravir is twice as fast, suggesting it has the potential to reduce VT risk in mothers who initiate ART late in pregnancy [41, 42, 43]. In the future, long‐acting agents administered during pregnancy/breastfeeding may further improve viral suppression, providing even greater benefits to mothers and infants [44].

### 4.1 Study limitations

We used strict laboratory/pharmacy criteria to infer HIV diagnoses in infants and so may have underestimated VT incidence. We used HIV diagnosis as the outcome of interest but the actual timing of acquisition could not be determined. Testing occurs at times specified programmatically and/or when there is clinical suspicion of HIV acquisition. Untreated, HIV in infants is usually characterized by rapid disease progression [45], with a typical time to symptomatic disease of several months and >50% mortality within 2 years [46], so it is reasonable to assume that the majority of children with HIV acquisition would have presented to health services during study follow‐up with symptoms and been tested (at study closure, median age of alive Group 1 infants without HIV diagnosis was 32 months; IQR 24–41). However, programmatic testing was incomplete, and the rate of disease progression can vary, therefore, it is likely that VT was underestimated. In models, among surviving 2‐ and 5‐year‐old children with HIV in South Africa in 2018, the projected proportion in whom HIV remained undiagnosed was 44% and 29%, respectively [47]. Almost all births in South Africa (96%) occur in health facilities [48] but across other sub‐Saharan African countries, as many as 78% of births occur outside health facilities [49]. In such settings, large gaps likely exist in services to prevent VT, and identifying children with HIV acquisition is more challenging.

Maternal data were unavailable for 25% of infants with HIV (Group 3); possibly because delivery facilities failed to perform electronic linkage when generating infant identifiers or because infants were born in other provinces or outside health facilities. Inter‐provincial mobility is relatively common and as the PHDC does not receive data from other provinces, we may have missing data, including infant HIV diagnoses/deaths. While most facilities in the province have electronic dispensing, there may be some missing pharmacy data. Substantial PNP data are likely missing as infant PNP dispensed after delivery is not always captured electronically, particularly at primary‐care delivery facilities. We, therefore, could not closely examine PNP effects.

Breastfeeding duration is a major determinant of postnatal VT [50] but breastfeeding data were not available. Because breastfeeding data, PNP data and infant HIV testing at ≥6 weeks were incomplete, the level of certainty at study closure that HIV‐exposed children were uninfected ranged from moderate (76%) to low (17%) to no certainty (7%), based on whether infants had any negative test at ≥6 weeks; a negative HIV‐PCR at <6 weeks but were not tested at ≥6 weeks; or were never tested, respectively (DECIPHER definitions) [51]. A high level of certainty requires more detailed information on the timing of tests at ≥6 weeks in relation to cessation of breastfeeding and PNP duration.

## 5 CONCLUSIONS

Despite high maternal ART coverage, ongoing VT is concerning. Trends in age at infant diagnosis suggest that most transmission occurs intrapartum and/or from breastfeeding. Interventions to improve maternal viral suppression are needed to reduce VT and achieve an HIV‐free generation.

## COMPETING INTERESTS

KA, M‐AD and EK received funding from ViiV Healthcare for this project. The other authors have no competing interests to declare.

## AUTHORS’ CONTRIBUTIONS

M‐AD contributed to study conception and design; AH performed data extraction; KA performed data analysis and drafted the manuscript; RK contributed insight into statistical aspects; all authors reviewed and approved the manuscript before submission.

## FUNDING

KA, M‐AD and EK received funding from ViiV Healthcare for this project. The PHDC was supported by Grant Number U01AI069924 from NIH (NIAID, NICHD, NCI, NIDA, NIMH)—PI: M. Egger and M‐A. Davies. AB, M‐AD and EK were supported by Grant Number R01HD080465 from NIH (NICHD); PI: A. Boulle. AB, AH and NJ were supported by Bill and Melinda Gates Foundation for the Measurement and Surveillance of HIV epidemics consortium (OPP1120138); Grant holder: J. Hargreaves.

## Supporting information


**Figure S1**: Number of HIV tests performed in group 1 infants, by week of life, during year 1 of life (n = 50 461 infants HIV‐exposed, whose mothers were diagnosed with HIV by delivery)
**Table S1**: Maternal HIV diagnosis, antiretroviral therapy and comorbidity characteristics, stratified by group
**Table S2**: Completeness of HIV‐PCR testing coverage at different time periods for Group 1 infants (with mothers known with HIV by delivery date)
**Table S3**: Poisson regression models assessing unadjusted associations with vertical transmission in infants whose mothers were known with HIV by delivery (Group 1 infants). Associations with HIV diagnosis in infants at age ≤7 days (N = 48 794), 8–98 days (N = 48 151) and >98 days (N = 47 844) were examined separately
**Table S4**: Mixed‐effects Poisson regression models assessing associations with vertical transmission in infants whose mothers were known with HIV by delivery (Group 1 infants). Sensitivity Model D assesses associations with HIV diagnosis in infants at age ≤7 days (N = 40 475), Model E at 8–98 days (N = 28 100 groups) and Model F at age >98 days (N = 17 588 groups). Infants without a HIV test in the analysis interval were excluded from analyses (Models D, E and F) and those without a negative test in the previous interval were excluded (Models E and F)
**Table S5**: Mixed‐effects Poisson regression models assessing associations with adverse outcomes in infants whose mothers were known with HIV by delivery (Group 1 infants). Infant death or vertical transmission were regarded as a composite adverse outcome. Sensitivity Model G assesses associations with death/HIV diagnosis in infants at age ≤7 days (N = 47 107), Model H at 8–98 days (N = 46 531 groups) and Model I at age >98 days (N = 46 269 groups)

## Data Availability

The data that support the findings of this study are available on request from the corresponding author. The data are not publicly available due to privacy or ethical restrictions.
