# Supplementary material for: Factors associated with vertical transmission of HIV in the Western Cape, South Africa: a retrospective cohort analysis
Source: J Int AIDS Soc. 2024 Mar 25;27(3):e26235. doi: 10.1002/jia2.26235 (PMC10963590; doi:10.1002/jia2.26235)
Supplement: Supplementary file 1 — Figure S1: Number of HIV tests performed in group 1 infants, by week of life, during year 1 of life (n = 50 461 infants HIV‐exposed, whose mothers were diagnosed with HIV by delivery) Table S1: Maternal HIV diagnosis, antiretroviral therapy and comorbidity characteristics, stratified by group Table S2: Completeness of HIV‐PCR testing coverage at different time periods for Group 1 infants (with mothers known with HIV by delivery date) Table S3: Poisson regression models assessing unadjusted associations with vertical transmission in infants whose mothers were known with HIV by delivery (Group 1 infants). Associations with HIV diagnosis in infants at age ≤7 days (N = 48 794), 8–98 days (N = 48 151) and >98 days (N = 47 844) were examined separately Table S4: Mixed‐effects Poisson regression models assessing associations with vertical transmission in infants whose mothers were known with HIV by delivery (Group 1 infants). Sensitivity Model D assesses associations with HIV diagnosis in infants at age ≤7 days (N = 40 475), Model E at 8–98 days (N = 28 100 groups) and Model F at age >98 days (N = 17 588 groups). Infants without a HIV test in the analysis interval were excluded from analyses (Models D, E and F) and those without a negative test in the previous interval were excluded (Models E and F) Table S5: Mixed‐effects Poisson regression models assessing associations with adverse outcomes in infants whose mothers were known with HIV by delivery (Group 1 infants). Infant death or vertical transmission were regarded as a composite adverse outcome. Sensitivity Model G assesses associations with death/HIV diagnosis in infants at age ≤7 days (N = 47 107), Model H at 8–98 days (N = 46 531 groups) and Model I at age >98 days (N = 46 269 groups) [file JIA2-27-e26235-s001.docx]

**Supplementary Material**

**Figure S1. Number of HIV tests performed in group 1 infants, by week of life, during year 1 of life (n=50 461 infants HIV-exposed, whose mothers were diagnosed with HIV by delivery)**

Note: tests include positive, negative and repeat tests (PCRs, antigen/antibody tests and available Rapids), but excludes duplicate results from the same day for the same infant. Rapid tests are not routinely captured electronically by all facilities and may be missing. Observed peaks in testing align with programmatic HIV-PCR testing at birth, 10 weeks and 6 months (and a small peak at 9 months due to programmatic Rapid screening which was conducted at 9 months at the start of the study period, prior to the introduction of 6 month HIV-PCR testing). In the first week of life (≤7 days), 98% of tests were infants’ first tests; during week 2 to 13 of life (day 8-98), 10% of total tests were infants’ first tests; and during week 14 to 51 of life (day 99-365), 2% of total tests were infants’ first tests.

**Table S1. Maternal HIV diagnosis, antiretroviral therapy and comorbidity characteristics, stratified by group**

|  | | Total | Group 1 | | 1A | | 1B | | Group 2 | |  |  |
| --- | --- | --- | --- | --- | --- | --- | --- | --- | --- | --- | --- | --- |
|  | |  | **Child HIV exposed,**  **maternal HIV evidence**  **≤delivery date** | | **Child HIV-negative, exposed to HIV** | | **Child with HIV** | | **Child with HIV, maternal HIV evidence**  **>delivery date** | |  |  |
| Timing of maternal HIV diagnosis, per infant (n=50764(50461;303)) | Before pregnancy | 36940 (72.2%) | | 36940 (73.2%) | | 36341 (73.3%) | | 599 (67.0%) | | 0 | |  |
|  | During pregnancy and <delivery date) | 12886 (25.2%) | | 12886 (25.5%) | | 12626 (25.5%) | | 260 (29.1%) | | 0 | |  |
|  | At delivery (on delivery date) | 635 (1.2%) | | 635 (1.3%) | | 600 (1.2%) | | 35 (3.9%) | | 0 | |  |
|  | After pregnancy (>delivery date) | 303 (0.6%) | | 0 | | 0 | | 0 | | 303 (100%) | |  |
| Detailed categories of maternal ART (combining ART start timing and ART gaps >14 days during pregnancy), per infant (n=50764(50461;303)) | Started before pregnancy and no gaps during pregnancy | 11415 (22.5%) | 11415 (22.6%) | | 11383 (23.0%) | | 32 (3.6%) | | 0 | |  |  |
|  | Started before pregnancy and had gap/s during pregnancy | 11639 (22.9%) | 11639 (23.1%) | | 11521 (23.2%) | | 118 (13.2%) | | 0 | |  |  |
|  | Started before pregnancy but no ART during pregnancy | 2438 (4.8%) | 2438 (4.8%) | | 2323 (4.7%) | | 115 (12.9%) | | 0 | |  |  |
|  | Started during pregnancy, >8 weeks before delivery, and no gap/s thereafter during pregnancy | 6614 (13.0%) | 6614 (13.1%) | | 6548 (13.2%) | | 66 (7.4%) | | 0 | |  |  |
|  | Started during pregnancy, >8 weeks before delivery, and had gaps thereafter during pregnancy | 5075 (10.0%) | 5075 (10.1%) | | 4950 (10.0%) | | 125 (14.0%) | | 0 | |  |  |
|  | Started during pregnancy, but within 8 weeks of delivery | 1754 (3.5%) | 1754 (3.5%) | | 1663 (3.4%) | | 91 (10.2%) | | 0 | |  |  |
|  | Restarted during pregnancy, > 8 weeks before delivery, and no gap/s thereafter during pregnancy | 1098 (2.2%) | 1098 (2.2%) | | 1075 (2.2%) | | 23 (2.6%) | | 0 | |  |  |
|  | Restarted during pregnancy, >8 weeks before delivery, and had gaps thereafter during pregnancy | 6131 (12.1%) | 6131 (12.1%) | | 5934 (12.0%) | | 197 (22.0%) | | 0 | |  |  |
|  | Restarted during pregnancy, but within 8 weeks of delivery | 1358 (2.7%) | 1358 (2.7%) | | 1303 (2.6%) | | 55 (6.2%) | | 0 | |  |  |
|  | No ART recorded prior to or during pregnancy | 3112 (6.1%) | 2809 (5.6%) | | 2738 (5.5%) | | 71 (7.9%) | | 303 (100%) | |  |  |
|  | Started during pregnancy but details unknown | 130 (0.3%) | 130 (0.3%) | | 129 (0.3%) | | 1 (0.1%) | | 0 | |  |  |
| Mother had any ART after delivery (≥infant DOB and up to 12 months post-delivery), per infant  (n=50764(50461;303)) | Yes and no gaps in ART of >2 weeks | 12747 (25.1%) | 12703 (25.2%) | | 12631 (25.5%) | | 72 (8.1%) | | N/A | |  |  |
|  | Yes but had gaps in ART of >2 weeks | 30669 (60.4%) | 30589 (60.6%) | | 29922 (60.4%) | | 667 (74.6%) | | N/A | |  |  |
|  | Yes but details unknown | 11 (0.02%) | 10 (0.02%) | | 9 (0.02%) | | 1 (0.1%) | | 0 | |  |  |
|  | No ART recorded | 7337 (14.5%) | 7159 (14.2%) | | 7005 (14.1%) | | 154 (17.2%) | | 178 (58.7%) | |  |  |
|  | Yes but only diagnosed and started after delivery | 0 | 0 | | 0 | | 0 | | 125 (41.3%) | |  |  |
| Evidence of maternal comorbidities, per infant (n=50764(50461;303)) | Diabetes | 1728 (3.4%) | 1721 (3.4%) | | 1702 (3.4%) | | 19 (2.1%) | | 7 (2.3%) | |  |  |
|  | Chronic kidney disease | 197 (0.4%) | 197 (0.4%) | | 188 (0.4%) | | 9 (1.0%) | | 0 | |  |  |
|  | Chronic obstructive pulmonary disease | 1592 (3.1%) | 1587 (3.1%) | | 1549 (3.1%) | | 38 (4.3%) | | 5 (1.7%) | |  |  |
|  | Hypertension | 4969 (9.8%) | 4951 (9.8%) | | 4867 (9.8%) | | 84 (9.4%) | | 18 (5.9%) | |  |  |
|  | Hypertensive disorder of pregnancy | 4455 (8.8%) | 4431 (8.8%) | | 4374 (8.8%) | | 57 (6.4%) | | 24 (7.9%) | |  |  |
|  | TB episode, started prior to pregnancy^†^ | 5819 (11.5%) | 5813 (11.5%) | | 5672 (11.4%) | | 141 (15.8%) | | 6 (2.0%) | |  |  |
|  | TB episode, started during pregnancy | 831 (1.6%) | 825 (1.6%) | | 791 (1.6%) | | 34 (3.8%) | | 6 (2.0%) | |  |  |
|  | TB episode, started after pregnancy | 948 (1.9%) | 942 (1.9%) | | 876 (1.8%) | | 66 (7.4%) | | 6 (2.0%) | |  |  |
| Note: denominators are indicated as ‘n=total(x;y)’ where appropriate, with x and y representing denominators for groups 1 and 2, respectively  Abbreviations: ART antiretroviral therapy; DOB date of birth; TB tuberculosis  ^†^Started ever before pregnancy (not necessarily ongoing during pregnancy) | | | | | | | | | | |  |  |

**Table S2. Completeness of HIV-PCR testing coverage at different time periods for Group 1 infants (with mothers known with HIV by delivery date)**

|  | Time period | N (%) |
| --- | --- | --- |
| Number of infants with a birth HIV-PCR (≤7 days old), by year of infant birth (n=50461(15051;15247;20163)) | Year 1 (01/05/2018 to 30/04/2019) | 13756 (91.4%) |
|  | Year 2 (01/05/2019 to 30/04/2020) | 13367 (87.7%) |
|  | Year 3 (01/05/2020 to 30/04/2021) | 16170 (80.2%) |
| Number of infants with a birth HIV-PCR, for infants born pre- vs post-lockdown^†^ (n=50461(28622;21839)) | Pre-lockdown | 25579 (89.4%) |
|  | Post-lockdown | 17714 (81.1%) |
| Number of infants^‡^ with a HIV-PCR at week 1-14 (day 8-98), by year of infant birth (n=14851;15087;19764) | Year 1 (01/05/2018 to 30/04/2019) | 10662 (71.8%) |
|  | Year 2 (01/05/2019 to 30/04/2020) | 9613 (63.7%) |
|  | Year 3 (01/05/2020 to 30/04/2021) | 13088 (66.2%) |
| Number of infants^‡^ with a HIV-PCR at week 1-14, pre^§^- vs post-lockdown (n=25380;24322) | Pre-lockdown | 17101 (67.4%) |
|  | Post-lockdown | 16262 (66.9%) |
| Number of infants^¶^ with a HIV-PCR at >98 days, by year of infant birth (n=14737;14967;19623) | Year 1 (01/05/2018 to 30/04/2019) | 4191 (28.4%) |
|  | Year 2 (01/05/2019 to 30/04/2020) | 7193 (48.1%) |
|  | Year 3 (01/05/2020 to 30/04/2021) | 12256 (62.5%) |

^†^South Africa introduced strict COVID-19 lockdown measures on 27 March 2020, which may have affected service delivery

^‡^Excluding infants with HIV diagnosis at age ≤7 days and those who died at age ≤7 days

^§^Included infants born 10 weeks before lockdown (i.e. before 17 January 2020)

^¶^Excluding infants with HIV diagnosis at age ≤98 days and those who died at age ≤98 days

Note: Completeness of birth HIV-PCR testing declined pre- vs post-lockdown (89% vs 81%) but completeness of testing at week 1-14 was unchanged (67%). It was not meaningful to compare testing at 6 months pre- vs post-lockdown because 6-month HIV-PCR testing was only introduced in 2020, hence the observed increase in Year 3.

**Table S3. Poisson regression models assessing unadjusted associations with vertical transmission in infants whose mothers were known with HIV by delivery (Group 1 infants). Associations with HIV diagnosis in infants at age ≤7 days (N=48 794), 8-98 days (N=48 151) and >98 days (N=47 844) were examined separately.**

|  | | HIV detected age ≤7 days | | | HIV detected age 8-98 days | | | | HIV detected age >98 days | | |
| --- | --- | --- | --- | --- | --- | --- | --- | --- | --- | --- | --- |
|  | | **IRR** | **95% CI** | **p** | **IRR** | **95% CI** | **p** | | **IRR** | **95% CI** | **p** |
| Infant sex male (vs female) | | 0.83 | (0.68-1.02) | 0.08 | 0.87 | (0.63-1.21) | | 0.40 | 1.20 | (0.98-1.48) | 0.09 |
| Low birthweight (<2500g) | | 3.05 | (2.46-3.79) | <0.001 | 1.76 | (1.19-2.60) | | 0.004 | 2.14 | (1.69-2.72) | <0.001 |
| Infant born before arrival at a health facility (vs born in a health facility) | | 1.71 | (0.96-3.04) | 0.07 | 3.01 | (1.48-6.15) | | 0.002 | 2.33 | (1.39-3.91) | 0.001 |
| Infant prophylaxis dispensed (within week 1 of life) | | N/A |  |  | 0.67 | (0.48-0.94) | | 0.02 | 1.05 | (0.85-1.29) | 0.64 |
| Maternal age category at delivery (years) | ≥ 30 years | 1 |  |  | 1 |  | |  | 1 |  |  |
|  | ≥ 20 years and <30 years | 1.55 | (1.26-1.92) | <0.001 | 1.65 | (1.17-2.32) | | 0.004 | 1.42 | (1.14-1.75) | 0.001 |
|  | < 20 years | 1.95 | (1.16 -3.26) | 0.01 | 2.28 | (1.04-5.01) | | 0.04 | 2.36 | (1.48-3.76) | <0.001 |
| Parity | 0 (primiparous) | 1 |  |  | 1 |  | |  | 1 |  |  |
|  | 1 | 0.90 | (0.71-1.14) | 0.37 | 0.91 | (0.62-1.33) | | 0.62 | 0.99 | (0.77-1.27) | 0.93 |
|  | ≥2 | 1.03 | (0.79-1.34) | 0.83 | 1.08 | (0.71-1.64) | | 0.72 | 1.45 | (1.13-1.88) | 0.004 |
| Mother likely acquired HIV vertically (HIV evidence <age 12 years vs later) | | 0.46 | (0.06-3.29) | 0.44 | 3.65 | (1.16-11.44) | | 0.03 | 3.30 | (1.47-7.39) | 0.004 |
| Maternal healthcare encounters during pregnancy | No gap in care of >3 months | 1 |  |  | 1 |  | |  | 1 |  |  |
|  | Gap/s in care of >3 months | 4.10 | (3.20-5.26) | <0.001 | 4.07 | (2.75-6.03) | | <0.001 | 3.11 | (2.46-3.94) | <0.001 |
|  | No care recorded | 12.19 | (8.03-18.53) | <0.001 | 8.17 | (3.77-17.68) | | <0.001 | 13.50 | (9.15-19.92) | <0.001 |
| Maternal healthcare encounters during postpartum (including delivery date and up until 12 months postpartum) | No gap in care of >3 months | N/A |  |  | 1 |  | |  | 1 |  |  |
|  | Gap/s in care of >3 months |  |  |  | 1.78 | (1.23-2.59) | | 0.002 | 3.52 | (2.65-4.67) | <0.001 |
|  | No care recorded |  |  |  | 1.43 | (0.51-4.02) | | 0.49 | 10.11 | (6.62-15.44) | <0.001 |
| Maternal HIV diagnosis | Before pregnancy | 1 |  |  | 1 |  | |  | 1 |  |  |
|  | During pregnancy | 1.70 | (1.36-2.11) | <0.001 | 1.15 | (0.80-1.66) | | 0.45 | 0.86 | (0.68-1.10) | 0.23 |
|  | At delivery | 6.27 | (4.08-9.63) | <0.001 | 1.79 | (0.57-5.63) | | 0.32 | 2.08 | (0.98-4.41) | 0.06 |
| Maternal ART started | Before pregnancy | 1 |  |  | 1 |  | |  | 1 |  |  |
|  | During pregnancy | 2.66 | (2.05-3.46) | <0.001 | 2.03 | (1.32-3.10) | | 0.001 | 1.40 | (1.07-1.82) | 0.01 |
|  | Restarted during pregnancy | 3.16 | (2.38-4.18) | <0.001 | 3.41 | (2.24-5.19) | | <0.001 | 3.20 | (2.49-4.11) | <0.001 |
|  | After pregnancy (>delivery date) | 11.59 | (7.56-17.78) | <0.001 | 5.05 | (2.00-12.78) | | 0.001 | 2.47 | (1.15-5.28) | 0.02 |
|  | No ART recorded | 1.37 | (0.73-2.55) | 0.32 | 1.73 | (0.73-4.07) | | 0.21 | 1.41 | (0.78-2.55) | 0.26 |
| For those who (first) started during pregnancy, ART start was | Trimester 1 | 1 |  |  | 1 |  | |  | 1 |  |  |
|  | Trimester 2 | 1.67 | (0.94-2.96) | 0.08 | 0.97 | (0.47-1.99) | | 0.93 | 1.29 | (0.77-2.16) | 0.33 |
|  | Trimester 3 | 5.52 | (3.11-9.81) | <0.001 | 1.31 | (0.54-3.15) | | 0.55 | 2.07 | (1.14-3.76) | 0.02 |
| Maternal ART during pregnancy (<infant DOB) | Yes and no gaps in ART of >2 weeks | 1 |  |  | 1 |  | |  | 1 |  |  |
|  | Yes but had gaps in ART of >2 weeks | 1.64 | (1.28-2.11) | <0.001 | 3.08 | (1.97-4.80) | | <0.001 | 2.53 | (1.96-3.28) | <0.001 |
|  | No ART recorded | 4.42 | (3.33-5.86) | <0.001 | 5.01 | (2.95-8.51) | | <0.001 | 4.56 | (3.30-6.31) | <0.001 |
| Maternal ART after delivery (≥infant DOB and up to 12 months post-delivery) | Yes and no gaps in ART of >2 weeks | N/A |  |  | 1 |  | |  | 1 |  |  |
|  | Yes but had gaps in ART of >2 weeks |  |  |  | 5.35 | (2.71-10.54) | | <0.001 | 4.04 | (2.70-6.04) | <0.001 |
|  | No ART recorded |  |  |  | 4.23 | (1.92-9.28) | | <0.001 | 6.67 | (4.28-10.41) | <0.001 |
| Detailed categories of antenatal maternal ART (combining ART start timing and ART dispensing gaps of >2 weeks in pregnancy) | Started before pregnancy and no gaps during pregnancy | 1 |  |  | 1 |  | |  | 1 |  |  |
|  | Started before pregnancy and had gap/s during pregnancy | 5.63 | (2.52-12.57) | <0.001 | 4.69 | (1.59-13.77) | | 0.01 | 2.71 | (1.62-4.51) | <0.001 |
|  | Started before pregnancy but no ART during pregnancy | 37.27 | (16.87-82.37) | <0.001 | 24.71 | (8.36-73.02) | | <0.001 | 16.58 | (9.82-27.97) | <0.001 |
|  | Started during pregnancy, more than 8 weeks before delivery, and no gap/s thereafter during pregnancy | 6.71 | (2.92-15.41) | <0.001 | 5.22 | (1.68-16.17) | | 0.004 | 1.97 | (1.10-3.55) | 0.02 |
|  | Started during pregnancy, more than 8 weeks before delivery, and had gaps thereafter during pregnancy | 15.88 | (7.20-35.07) | <0.001 | 13.08 | (4.52-37.82) | | <0.001 | 5.45 | (3.24-9.15) | <0.001 |
|  | Started during pregnancy, but within 8 weeks of delivery | 54.96 | (25.07-120.49) | <0.001 | 15.35 | (4.73-49.85) | | <0.001 | 7.89 | (4.33-14.37) | <0.001 |
|  | Restarted during pregnancy, more than 8 weeks before delivery, and no gap/s thereafter during pregnancy | 13.50 | (5.03-36.26) | <0.001 | 5.28 | (0.97-28.83) | | 0.06 | 5.47 | (2.67-11.19) | <0.001 |
|  | Restarted during pregnancy, more than 8 weeks before delivery, and had gaps thereafter during pregnancy | 18.61 | (8.55-40.48) | <0.001 | 15.61 | (5.53-44.06) | | <0.001 | 10.01 | (6.18-16.23) | <0.001 |
|  | Restarted during pregnancy, but within 8 weeks of delivery | 28.38 | (12.18-66.15) | <0.001 | 26.20 | (8.45-81.22) | | <0.001 | 8.40 | (4.40-16.04) | <0.001 |
|  | No ART recorded prior to or during pregnancy | 22.61 | (10.10-50.64) | <0.001 | 11.50 | (3.66-36.11) | | <0.001 | 4.73 | (2.52-8.86) | <0.001 |
| Regimen prior to delivery | NNRTI-based | 1 |  |  | 1 |  | |  | 1 |  |  |
|  | PI-based | 2.32 | (1.54-3.49) | <0.001 | 2.29 | (1.26-4.18) | | 0.01 | 1.57 | (1.01-2.43) | 0.04 |
|  | INSTI-based | 1.51 | (1.07-2.12) | 0.02 | 0.65 | (0.31-1.33) | | 0.24 | 1.31 | (0. 84-2.04) | 0.24 |
| YEAR of infant birth | Year 1 (01/05/2018 to 30/04/2019) | 1 |  |  | 1 |  | |  | 1 |  |  |
|  | Year 2 (01/05/2019 to 30/04/2020) | 0.86 | (0.66-1.10) | 0.23 | 0.97 | (0.64-1.46) | | 0.88 | 1.42 | (1.11-1.82) | 0.01 |
|  | Year 3 (01/05/2020 to 30/04/2021) | 0.94 | (0.74-1.20) | 0.63 | 1.05 | (0.70-1.56) | | 0.83 | 2.71 | (2.10-3.50) | <0.001 |
| VL nearest delivery (copies/ml) (within 1 month before pregnancy start and up to 7 days after delivery) | <100 | 1 |  |  | 1 |  | |  | 1 |  |  |
|  | 100-999 | 9.03 | (5.42-15.04) | <0.001 | 7.11 | (3.42-14.77) | | <0.001 | 4.46 | (3.13-6.35) | <0.001 |
|  | 1000-99999 | 38.89 | (26.15-57.82) | <0.001 | 25.45 | (14.58-44.41) | | <0.001 | 10.09 | (7.65-13.31) | <0.001 |
|  | ≥100000 | 115.78 | (74.58-179.74) | <0.001 | 86.19 | (46.02-161.42) | | <0.001 | 28.25 | (19.48-40.98) | <0.001 |
|  | Unknown | 23.35 | (15.38-35.44) | <0.001 | 15.77 | (8.70-28.57) | | <0.001 | 5.63 | (4.13-7.67) | <0.001 |
| Time-updated VL (copies/ml) | <100 | 1 |  |  | 1 |  | |  | 1 |  |  |
|  | 100-999 | 8.96 | (5.33-15.06) | <0.001 | 10.10 | (4.81-21.23) | | <0.001 | 4.65 | (2.26-9.53) | <0.001 |
|  | 1000-99999 | 35.61 | (23.72-53.47) | <0.001 | 33.78 | (18.49-61.69) | | <0.001 | 12.73 | (7.85-20.64) | <0.001 |
|  | ≥100000 | 109.75 | (69.91-172.30) | <0.001 | 108.17 | (55.59-210.50) | | <0.001 | 57.55 | (35.59-93.07) | <0.001 |
|  | Unknown | 25.61 | (16.95-38.70) | <0.001 | 14.86 | (7.80-28.31) | | <0.001 | 4.60 | (3.13-6.75) | <0.001 |
| Immune deficiency category nearest delivery (per CD4 count; cells/µl) (from 15 months before delivery to 7 days after) | No deficiency (≥500) | 1 |  |  | 1 |  | |  | 1 |  |  |
|  | Mild (350-499) | 2.18 | (1.53-3.13) | <0.001 | 2.14 | (1.07-4.26) | | 0.03 | 2.03 | (1.32-3.12) | 0.001 |
|  | Advanced (200-349) | 2.64 | (1.87-3.75) | <0.001 | 4.31 | (2.33-7.96) | | <0.001 | 3.52 | (2.39-5.21 ) | <0.001 |
|  | Severe (<200) | 6.17 | (4.44-8.59) | <0.001 | 8.03 | (4.37-14.75) | | <0.001 | 9.16 | (6.32-13.26) | <0.001 |
|  | Unknown | 0.89 | (0.60-1.32) | 0.56 | 2.14 | (1.15-4.00) | | 0.02 | 2.23 | (1.51-3.29 ) | <0.001 |
| Time-updated CD4 immune deficiency category (per CD4 count; cells/µl) | No deficiency (≥500) | 1 |  |  | 1 |  | |  | 1 |  |  |
|  | Mild (350-499) | 2.20 | (1.52-3.19) | <0.001 | 1.89 | (0.95-3.76) | | 0.07 | 3.65 | (1.96-6.80) | <0.001 |
|  | Advanced (200-349) | 2.67 | (1.86-3.81) | <0.001 | 4.21 | (2.32-7.64) | | <0.001 | 9.38 | (5.41-16.29) | <0.001 |
|  | Severe (<200) | 6.15 | (4.38-8.64) | <0.001 | 7.88 | (4.37-14.21) | | <0.001 | 16.76 | (9.78-28.71) | <0.001 |
|  | Unknown | 1.06 | (0.73-1.54) | 0.76 | 1.51 | (0.81-2.80) | | 0.20 | 1.98 | (1.19-3.30) | 0.01 |
| Age category | Age 3 to 5 months |  |  |  |  |  | |  | 1 |  |  |
|  | Age ≥5 to 8 months |  |  |  |  |  | |  | 0.49 | (0.34-0.71) | <0.001 |
|  | Age ≥8 to 11 months |  |  |  |  |  | |  | 0.47 | (0.31-0.70) | <0.001 |
|  | Age ≥11 to 14 months |  |  |  |  |  | |  | 0.41 | (0.26-0.64) | <0.001 |
|  | Age ≥14 to 17 months |  |  |  |  |  | |  | 0.40 | (0.25-0.62) | <0.001 |
|  | Age ≥17 months to 24 months |  |  |  |  |  | |  | 0.19 | (0.13-0.28) | <0.001 |
|  | Age ≥24 months |  |  |  |  |  | |  | 0.05 | (0.03-0.07) | <0.001 |

Abbreviations: IRR incidence rate ratio; CI confidence interval; ART antiretroviral therapy; DOB date of birth; NNRTI nonnucleoside reverse transcriptase inhibitor; PI protease inhibitor; INSTI integrase strand transfer inhibitors; VL viral load

**Table S4. Mixed-effects Poisson regression models assessing associations with vertical transmission in infants whose mothers were known with HIV by delivery (Group 1 infants). Sensitivity Model D assesses associations with HIV diagnosis in infants at age ≤7 days (N=40 475), Model E at 8-98 days (N=28 100 groups) and Model F at age >98 days (N=17 588 groups). Infants without a HIV test in the analysis interval were excluded from analyses (Models D ,E and F) and those without a negative test in the previous interval were excluded (Models E and F).**

|  | | Model D | | | | Model E | | | | Model F | | |
| --- | --- | --- | --- | --- | --- | --- | --- | --- | --- | --- | --- | --- |
|  | | **aIRR** | **95% CI** | **p** | **aIRR** | | **95% CI** | **p** | **aIRR** | | **95% CI** | **p** |
| Infant sex male (vs female) | | 0.75 | (0.55-1.01) | 0.06 | 0.99 | | (0.67-1.48) | 0.98 | 1.37 | | (1.03-1.81) | 0.03 |
| Low birthweight (<2500g vs ≥2500g) | | 2.79 | (1.97-3.96) | <0.001 | 1.03 | | (0.63-1.68) | 0.92 | 1.28 | | (0.91-1.78) | 0.15 |
| Infant prophylaxis dispensed within week 1 of life (vs none recorded) | | N/A |  |  | 0.83 | | (0.56-1.25) | 0.38 | 1.13 | | (0.85-1.50) | 0.39 |
| Maternal age category at delivery (years) | ≥ 30 years | 1 |  |  | 1 | |  |  | 1 | |  |  |
|  | ≥ 20 years and <30 years | 1.18 | (0.85-1.64) | 0.33 | 1.00 | | (0.65-1.53) | 0.98 | 1.44 | | (1.06-1.94) | 0.02 |
|  | < 20 years | 1.14 | (0.51-2.57) | 0.75 | 1.75 | | (0.73-4.20) | 0.21 | 2.07 | | (1.02-4.19) | 0.04 |
| Mother likely acquired HIV vertically infected evidence <age 12 years vs later) | | 0.15 | (0.01-2.04) | 0.16 | 2.28 | | (0.65-7.95) | 0.20 | 1.67 | | (0. 50-5.55) | 0.41 |
| Parity | 0 (primiparous) | 1 |  |  | 1 | |  |  | 1 | |  |  |
|  | 1 | 1.01 | (0.69-1.45) | 0.98 | 0.78 | | (0.47-1.31) | 0.35 | 1.06 | | (0.75-1.51) | 0.73 |
|  | ≥2 | 1.16 | (0.75-1.80) | 0.49 | 1.12 | | (0.65-1.94) | 0.68 | 1.55 | | (1.07-2.24) | 0.02 |
| Detailed categories of antenatal maternal ART (combining ART start timing and ART dispensing gaps of >2 weeks during pregnancy) | Started before pregnancy and no ART gaps of during pregnancy | 1 |  |  | 1 | |  |  | 1 | |  |  |
|  | Started before pregnancy and had gap/s during pregnancy | 3.76 | (1.45-9.79) | 0.01 | 2.94 | | (0.84-10.29) | 0.09 | 2.33 | | (1.21-4.48) | 0.01 |
|  | Started before pregnancy but no ART during pregnancy | 11.35 | (4.23-30.47) | <0.001 | 2.80 | | (0.68-11.53) | 0.15 | 9.44 | | (4.49-19.82) | <0.001 |
|  | Started during pregnancy, more than 8 weeks before delivery, and no gap/s thereafter during pregnancy | 4.71 | (1.72-12.94) | 0.003 | 3.23 | | (0.85-12.35) | 0.09 | 1.65 | | (0.75-3.62) | 0.21 |
|  | Started during pregnancy, more than 8 weeks before delivery, and had gaps thereafter during pregnancy | 7.57 | (2.86-20.05) | <0.001 | 5.20 | | (1.46-18.53) | 0.01 | 4.84 | | (2.44-9.59) | <0.001 |
|  | Started during pregnancy, but within 8 weeks of delivery | 25.97 | (9.58-70.44) | <0.001 | 2.54 | | (0.55-11.68) | 0.23 | 5.91 | | (2.67-13.06) | <0.001 |
|  | Restarted during pregnancy, more than 8 weeks before delivery, and no gap/s thereafter during pregnancy | 10.32 | (2.85-37.42) | <0.001 | 1.98 | | (0.20-19.23) | 0.56 | 6.65 | | (2.74-16.16) | <0.001 |
|  | Restarted during pregnancy, more than 8 weeks before delivery, and had gaps thereafter during pregnancy | 9.22 | (3.58-23.75) | <0.001 | 5.51 | | (1.62-18.72) | 0.01 | 7.38 | | (3.99-13.66) | <0.001 |
|  | Restarted during pregnancy, but within 8 weeks of delivery | 7.08 | (2.44-20.55) | <0.001 | 7.58 | | (2.05-28.00) | 0.002 | 6.51 | | (2.80-15.14) | <0.001 |
|  | No ART recorded prior to or during pregnancy | 14.10 | (5.14-38.68) | <0.001 | 3.97 | | (0.97-16.18) | 0.06 | 4.86 | | (2.06-11.45) | <0.001 |
| Time-updated CD4 immune deficiency category (per CD4 count; cells/µl) | No deficiency (≥500) | 1 |  |  | 1 | |  |  | 1 | |  |  |
|  | Mild (350-499) | 1.66 | (0.99-2.80) | 0.06 | 1.67 | | (0.70-4.01) | 0.25 | 1.59 | | (0.71-3.54) | 0.26 |
|  | Advanced (200-349) | 1.26 | (0.76-2.10) | 0.36 | 2.37 | | (1.07-5.28) | 0.03 | 3.16 | | (1.58-6.30) | 0.001 |
|  | Severe (<200) | 2.76 | (1.64-4.63) | <0.001 | 2.63 | | (1.17-5.95) | 0.02 | 5.05 | | (2.59-9.87) | <0.001 |
|  | Unknown | 0.94 | (0.56-1.60) | 0.83 | 1.86 | | (0.81-4.30) | 0.14 | 2.80 | | (1.52-5.15) | 0.001 |
| Time-updated VL (copies/ml) | <100 | 1 |  |  | 1 | |  |  | 1 | |  |  |
|  | 100-999 | 7.35 | (3.97-13.60) | <0.001 | 11.83 | | (4.91-28.48) | <0.001 | 1.45 | | (0.50-4.26) | 0.50 |
|  | 1000-99999 | 38.38 | (23.03-63.97) | <0.001 | 26.69 | | (12.20-58.38) | <0.001 | 4.59 | | (2.45-8.57) | <0.001 |
|  | ≥100000 | 254.36 | (126.76-510.42) | <0.001 | 95.68 | | (40.54-225.85) | <0.001 | 22.33 | | (11.73-42.50) | <0.001 |
|  | Unknown | 34.93 | (20.53-59.41) | <0.001 | 13.59 | | (5.59-33.05) | <0.001 | 5.43 | | (3.33-8.74) | <0.001 |
| YEAR of infant birth | Year 1 (01/05/2018 to 30/04/2019) | 1 |  |  | 1 | |  |  | 1 | |  |  |
|  | Year 2 (01/05/2019 to 30/04/2020) | 1.13 | (0.78-1.65) | 0.51 | 0.85 | | (0.50-1.46) | 0.56 | 0.59 | | (0.41-0.83) | 0.003 |
|  | Year 3 (01/05/2020 to 30/04/2021) | 1.44 | (0.98-2.10) | 0.06 | 1.09 | | (0.68-1.75) | 0.71 | 0.34 | | (0.24-0.50) | <0.001 |

Abbreviations: aIRR adjusted incidence rate ratio; CI confidence interval; ART antiretroviral therapy; VL viral load

Adjusted IRRs (and 95% confidence intervals) were obtained from mixed-effects Poisson regression models (log link function; normally distributed random effect by mother; observation time as an offset). Estimated variance of random effects at maternal level were all < 0.01, suggesting no remaining unexplained inter-mother variability. Twins and triplets were excluded from analyses. In Model F, an age category covariate was included (age 3-5, 5-8, 8-11, 11-14, 14-17, 17-24 and ≥24 months).

**Table S5. Mixed-effects Poisson regression models assessing associations with adverse outcomes in infants whose mothers were known with HIV by delivery (Group 1 infants). Infant death or vertical transmission were regarded as a composite adverse outcome. Sensitivity Model G assesses associations with death/HIV diagnosis in infants at age ≤7 days (N=47 107), Model H at 8-98 days (N=46 531 groups) and Model I at age >98 days (N=46 269 groups).**

|  |  | |  |  |  | | | Model G | | | | | | | | | Model H | | | | | Model I | | | | | |
| --- | --- | --- | --- | --- | --- | --- | --- | --- | --- | --- | --- | --- | --- | --- | --- | --- | --- | --- | --- | --- | --- | --- | --- | --- | --- | --- | --- |
|  | | | | | | **aIRR** | | | | | **95% CI** | | **p** | | | **aIRR** | | **95% CI** | | **p** | **aIRR** | | | | **95% CI** | | **p** |
| Infant sex male (vs female) | | | | | | 0.93 | | | | | (0.75-1.15) | | 0.50 | | | 1.13 | | (0.87-1.48) | | 0.36 | 1.25 | | | | (1.04-1.49) | | 0.02 |
| Low birthweight (<2500g vs ≥2500g) | | | | | | 10.86 | | | | | (8.55-13.79) | | <0.001 | | | 6.45 | | (4.84-8.60) | | <0.001 | 1.71 | | | | (1.39-2.10) | | <0.001 |
| Infant prophylaxis dispensed within week 1 of life (vs none recorded) | | | | | | N/A | | | | |  | |  | | | 0.81 | | (0.62-1.07) | | 0.14 | 1.13 | | | | (0.94-1.36) | | 0.18 |
| Maternal age category at delivery (years) | | ≥ 30 years | | | | 1 | | | | |  | |  | | | 1 | |  | |  | 1 | | | |  | |  |
|  |  | ≥ 20 years and <30 years | | | | 1.19 | | | | | (0.95-1.50) | | 0.13 | | | 0.89 | | (0.66-1.18) | | 0.41 | 1.07 | | | | (0.89-1.30) | | 0.48 |
|  |  | < 20 years | | | | 0.93 | | | | | (0.49-1.79) | | 0.84 | | | 0.73 | | (0.32-1.70) | | 0.47 | 1.80 | | | | (1.15-2.80) | | 0.01 |
| Mother likely acquired HIV vertically (HIV evidence <age 12 years vs later) | | | | | | 0.49 | | | | | (0.10-2.47) | | 0.39 | | | 3.91 | | (1.16-13.14) | | 0.03 | 1.33 | | | | (0.57-3.10) | | 0.51 |
| Parity | | 0 (primiparous) | | | | 1 | | | | |  | |  | | | 1 | |  | |  | 1 | | | |  | |  |
|  |  | 1 | | | | 1.18 | | | | | (0.91-1.53) | | 0.22 | | | 1.03 | | (0.74-1.43) | | 0.86 | 1.01 | | | | (0.81-1.27) | | 0.90 |
|  |  | ≥2 | | | | 1.80 | | | | | (1.35-2.40) | | <0.001 | | | 1.31 | | (0.91-1.89) | | 0.14 | 1.16 | | | | (0.91-1.48) | | 0.24 |
| Detailed categories of antenatal maternal ART (combining ART start timing and ART dispensing gaps of >2 weeks during pregnancy) | | Started before pregnancy and no ART gaps of during pregnancy | | | | 1 | | | | |  | |  | | | 1 | |  | |  | 1 | | | |  | |  |
|  |  | Started before pregnancy and had gap/s during pregnancy | | | | 0.97 | | | | | (0.66-1.44) | | 0.90 | | | 1.06 | | (0.67-1.66) | | 0.81 | 1.59 | | | | (1.10-2.39) | | 0.01 |
|  |  | Started before pregnancy but no ART during pregnancy | | | | 1.86 | | | | | (1.13-3.06) | | 0.01 | | | 1.72 | | (0.93-3.17) | | 0.08 | 5.61 | | | | (3.70-8.51) | | <0.001 |
|  |  | Started during pregnancy, more than 8 weeks before delivery, and no gap/s thereafter during pregnancy | | | | 1.13 | | | | | (0.71-1.79) | | 0.61 | | | 0.90 | | (0.51-1.60) | | 0.72 | 1.29 | | | | (0.83-2.00) | | 0. 26 |
|  |  | Started during pregnancy, more than 8 weeks before delivery, and had gaps thereafter during pregnancy | | | | 1.36 | | | | | (0.85-2.16) | | 0.20 | | | 1.23 | | (0.71-2.14) | | 0.47 | 2.62 | | | | (1.76-3.91) | | <0.001 |
|  |  | Started during pregnancy, but within 8 weeks of delivery | | | | 3.90 | | | | | (2.34-6.50) | | <0.001 | | | 1.35 | | (0.67-2.73) | | 0.40 | 3.56 | | | | (2.20-5.74) | | <0.001 |
|  |  | Restarted during pregnancy, more than 8 weeks before delivery, and no gap/s thereafter during pregnancy | | | | 1.76 | | | | | (0.85-3.65) | | 0.13 | | | 1.50 | | (0.64-3.52) | | 0.35 | 2.51 | | | | (1.37-4.59) | | 0.003 |
|  |  | Restarted during pregnancy, more than 8 weeks before delivery, and had gaps thereafter during pregnancy | | | | 1.21 | | | | | (0.78-1.86) | | 0.40 | | | 1.14 | | (0.69-1.90) | | 0.61 | 3.48 | | | | (2.43-4.99) | | <0.001 |
|  |  | Restarted during pregnancy, but within 8 weeks of delivery | | | | 2.15 | | | | | (1.22-3.79) | | 0.01 | | | 1.62 | | (0.80-3.26) | | 0.18 | 3.17 | | | | (1.88-5.36) | | <0.001 |
|  |  | No ART recorded prior to or during pregnancy | | | | 1.89 | | | | | (1.15-3.10) | | 0.01 | | | 0.70 | | (0.34-1.45) | | 0.34 | 2.81 | | | | (1.72-4.59) | | <0.001 |
| Time-updated CD4 immune deficiency category (per CD4 count; cells/µl) | | No deficiency (≥500) | | | | 1 | | | | |  | |  | | | 1 | |  | |  | 1 | | | |  | |  |
|  |  | Mild (350-499) | | | | 1.25 | | | | | (0.88-1.78) | | 0.22 | | | 1.34 | | (0.83-2.16) | | 0.22 | 1.67 | | | | (1.09-2.56) | | 0.02 |
|  |  | Advanced (200-349) | | | | 1.10 | | | | | (0.77-1.56) | | 0.61 | | | 2.02 | | (1.30-3.15) | | 0.002 | 2.34 | | | | (1.59-3.47) | | <0.001 |
|  |  | Severe (<200) | | | | | 1.75 | | (1.20-2.53) | | | | | 0.003 | 2.19 | | | | (1.36-3.54) | 0.001 | | | 3.47 | (2. 36-5.11) | | <0.001 | |
|  |  | Unknown | | | | | 0.89 | | (0.65-1.24) | | | | | 0.50 | 1.28 | | | | (0.84-1.94) | 0.25 | | | 2.02 | (1.43-2.85) | | <0.001 | |
| Time-updated VL (copies/ml) | | <100 | | | | | 1 | |  | | | | |  | 1 | | | |  |  | | | 1 |  | |  | |
|  |  | 100-999 | | | | | 1.93 | | (1.28-2.92) | | | | | 0.002 | 1.83 | | | | (1.16-2.90) | 0.01 | | | 1.89 | (1.09-3.28) | | 0.02 | |
|  |  | 1000-99999 | | | | | 7.55 | | (5.48-10.40) | | | | | <0.001 | 3.94 | | | | (2.69-5.77) | <0.001 | | | 3.91 | (2.68-5.70) | | <0.001 | |
|  |  | ≥100000 | | | | | 34.43 | | (20.95-56.58) | | | | | <0.001 | 11.05 | | | | (5.78-21.12) | <0.001 | | | 14.65 | (9. 63-22.29) | | <0.001 | |
|  |  | Unknown | | | | | 6.24 | | (4.54-8.59) | | | <0.001 | | | 2.20 | | | | (1.47-3.30) | <0.001 | | | 4.72 | (3.56-6.27) | | <0.001 | |
| YEAR of infant birth | | Year 1 (01/05/2018 to 30/04/2019) | | | | | 1 | | |  | |  | | | 1 | | | |  |  | | | 1 |  | |  | |
|  |  | Year 2 (01/05/2019 to 30/04/2020) | | | | | 0.74 | | | (0.57-0.97) | | 0.03 | | | 0.82 | | | | (0.59-1.14) | 0.24 | | | 0.87 | (0.70-1.09) | | 0.22 | |
|  |  | Year 3 (01/05/2020 to 30/04/2021) | | | | | 1.06 | | | (0.82-1.38) | | 0.64 | | | 0.86 | | | | (0.62-1.20) | 0.38 | | | 0.96 | (0. 76-1.21) | | 0.74 | |

Abbreviations: aIRR adjusted incidence rate ratio; CI confidence interval; ART antiretroviral therapy; VL viral load

Adjusted IRRs (and 95% confidence intervals) were obtained from mixed-effects Poisson regression models (log link function; normally distributed random effect by mother; observation time as an offset). Twins and triplets were excluded from analyses. In Model I, an age category covariate was included (age 3-5, 5-8, 8-11, 11-14, 14-17, 17-24 and ≥24 months).
